# Conducting online OSCEs aided by a novel time management web-based system

**DOI:** 10.1186/s12909-021-02945-9

**Published:** 2021-09-26

**Authors:** Sami Shaban, Irfan Tariq, Margaret Elzubeir, Ahmed R Alsuwaidi, Alsajir Basheer, Mohi Magzoub

**Affiliations:** 1 Department of Medical Education, College of Medicine and Health Sciences, United Arab Emirates University, PO Box 17666, Al Ain, UAE; 2 Department of Pediatrics, College of Medicine and Health Sciences, United Arab Emirates University, Al Ain, UAE

**Keywords:** OSCE, Time Management System, Online OSCE, Web OSCE, Virtual OSCE, COVID-19

## Abstract

**Background:**

Objective Structured Clinical Examinations (OSCEs) are an essential part of the assessment process for medical students. They have traditionally been face-to-face assessments, however, due to the COVID-19 pandemic, medical schools have been forced to attempt to carry them out remotely. OSCEs are difficult to carry out online due to rotation management aspects which make it difficult to synchronize movement of students from one station to another.

**Methods:**

The authors have developed a dynamic OSCE time management website which aids in solving the movement synchronization issue. This secure website enables participants to view the list of stations they are allowed to enter, and the start and end time of each station. OSCE administrators can control time of entry and monitor progress of the OSCE remotely.

**Results:**

The authors have used the system to conduct several exams successfully, showing the feasibility and cost effectiveness of this method, as well as user acceptance and satisfaction. In contrast to traditional OSCEs, students are set up in individual virtual rooms for the whole exam while examiners, simulated patients and proctors rotate between them.

**Conclusions:**

This online OSCE implementation shows feasibility, cost effectiveness and acceptance of this method. The authors found that student outcomes are comparable to traditional OSCEs conducted in the past. There was no significant difference in student marks in one exam compared to last year, while marks were slightly higher in two exams, potentially due to lack of physical exam stations. An unresolved drawback is the inability to assess physical exam stations online, although having students verbally describe what they would do in physical exam situations may be a partial solution.

## Practice points

1. OSCEs are difficult to conduct online due to rotation management aspects which make it difficult to synchronize movement of students (or examiners and SPs ) from one station to another.
2. An OSCE time management website can aid in solving the movement synchronization issue.
3. Participants must be able to view the list of assigned stations along with start and end time.
4. OSCE administrators must be able to control time of entry and monitor progress of the OSCE remotely.

## Background

Objective Structured Clinical Examinations (OSCEs) are a well-established tool for providing authentic, simulated, performance-based assessments in pre- and post-registration health professions education [1, 2]. OSCEs also facilitate learner in-person practice prior to clinical placement, maximizing opportunities for high and low stakes assessment in a safe environment appropriate to learner stages of training. Hodges [3] describes the original OSCE examination as a series of highly structured, timed, scripted ‘scenes’ of standardized patient interactions across multiple domains with healthcare professional students. As such, for over three decades, OSCEs have represented the gold standard in medical student assessment and lend themselves to research relating to process, structure, outcomes and impact of the examination on health professions students’ performance.

However, although a versatile, multipurpose evaluative tool, based on principles of objectivity and standardization [4], organization and delivery of OSCEs in traditional in-person, clinical simulation environments can be a costly, resource intensive and a logistically challenging activity [5, 6]. Research into assessment cost-effectiveness, feasibility, reliability and acceptability, therefore, represents important indicators of OSCE utility [7, 8] in both traditional and non-traditional circumstances.

The trend toward online (virtual) or ‘non-traditional’ education and assessment is not new. Health professions educators have been using technology to overcome challenges when providing hands-on simulated training and assessment of communications, physical examination, diagnostic reasoning and management competencies and skills of medical students for some time [9–13]. However, the COVID-19 pandemic has necessitated an accelerated use of technology in education. Medical school leaders have had to rapidly adapt educational provision to safeguard the health of students, patients and faculty members while simultaneously assuring achievement of core competencies and quality standards in the assessment of these competencies. These unique challenges have required educational innovations and led health professions educators to adopt replacement of traditional in-person educational experiences with alternative teaching and assessment of students at a distance [9, 14].

In some institutions, clerkships, OSCEs and simulation-based in-person activities were initially scheduled with limitations on numbers of students per session. Subsequent guidelines from various accrediting agencies then emerged advising suspension of clerkships, finding other curricular opportunities where clerkship objectives could be achieved and transitioning to online learning as ways of facilitating students’ acquisition and assessment of clinical skills. Additionally, in many cases, high stakes exams were postponed, disrupting timely student graduation and requirements for advancement to the next stage of education and training. However, to mitigate the risk of significantly hindering students’ progression to internship, residency programs and clinical workforce, a few medical schools sought solutions for implementation of online OSCE formats while adopting strategies to minimize academic misconduct and preserve validity and reliability of the assessments.

Institutions in the region such as Weill Cornell in Qatar and Duke University of Singapore were among the forerunners publicizing their activities in this regard [15, 16], illustrating how online OSCEs can be a solution to unforeseen challenges while seizing opportunities to transform and reshape educational practices. Indeed, these few descriptions of how medical educators overcame confounding factors and logistical complexities of launching online OSCEs have been helpful to the medical education community. It is clear that new evidence-based assessment practices in response to the COVID-19 pandemic, their feasibility, acceptability and cost effectiveness are of importance in the current medical education climate.

In our institution, the MD Program is a 6-year undergraduate, high school entry program which admits approximately 100–150 Emirati national medical students per year. There are three two-year phases within the program which are PreMedical, PreClinical and Clinical. Students conduct OSCEs in the PreClinical and Clinical phases. Under normal circumstances, our MD students would take a total of 5 major summative OSCEs during the program and a few minor OSCEs during their clerkships.

Clinical skills development starts in the PreClinical system-based phase, which entails demonstration of required skills during practical hands-on sessions on manikins and interactions with simulated patients. Covered clinical skills include history taking, physical examination, communication skills including breaking bad news and counseling, and building clinical reasoning skills. During the pandemic, hybrid learning methods have been utilized which include some face-to-face learning in small groups as well as online demonstrations and discussions. Thus, our students are very familiar with eLearning and online resources for teaching and assessment made available to them.

Stations were reviewed as is normally done each year but since all stations entailed demonstration of history taking and communication skills they were basically similar to traditional face-to-face stations and easily transformed to online delivery.

### Study purpose and objectives

The aim of this study is to describe and evaluate one medical school’s implementation of high-stakes online OSCEs incorporating a novel time management system, applying four assessment utility indices (feasibility, cost effectiveness, acceptability and validity) [17] as a framework for our evaluation. The study was driven by the following objectives:

- To provide a step-by-step description of planning and carrying out of this online OSCE implementation conducted June/July 2020.
- To evaluate, from the perspective of designers and providers (administrators and faculty), feasibility and cost effectiveness of this online OSCE implementation.
- To evaluate, from perspective of students and faculty, acceptability of this online OSCE implementation and their reactions to it.
- To determine if there are significant differences in performance outcomes of students experiencing traditional face-to-face OSCEs compared to online OSCEs.

We have chosen to develop this method due to the lack of other software options that fulfill the needs of OSCE procedures and performance assessment.

## Methods

This online OSCE system was designed to test medical students on their history taking and communication skills while in remote settings. There are three main technologies involved:

1. Video conferences software, in our case Microsoft Teams (but it is possible to use other appropriate software).
2. Time synchronization software, in this case a locally developed OSCE Time Management Dynamic Website (to our knowledge, this is a novel idea and there is currently no other software for this task ).
3. Performance documenting software, in this case Speedwell used by examiners to document performance (but other methods can be used instead).

In contrast to traditional OSCEs, we set up each student in his or her individual virtual room on Teams for the entirety of the exam, and had examiners, simulated patients (SPs) and proctors rotating between them. This enabled monitoring and recording of students within and between stations. They were provided with a link to their virtual room by logging on to the OSCE time management website at the time of the examination. Examiners, SPs and proctors also logged on to this website at the beginning of the exam. The OSCE time management website acted as the guide for when they should enter and exit each virtual room. The website also provided a link to the correct virtual room. In addition, examiners used an examination software to enter their marking of student performance.

The system is designed for students to be in a remote location in a private, quiet, well-lit room, which has a computer with good internet connection (preferably wired) and a headset (microphone and speakers). Students are instructed to not have any other devices (e.g. mobile) in the room. Students each enter their own virtual room in which audio-visual recording is in progress the entire time (even between stations) and students stay in that virtual room from beginning to end of the OSCE exam. Examiners and SPs are the ones who rotate in and out of the virtual rooms.

Faculty, SPs, Proctors and OSCE Administrators can also be remote however in some OSCE examinations that we have conducted, they have chosen to be physically present in the College in isolated on-campus rooms with a computer connected to the Internet and examiners with an additional iPad for marking. The system was tested using one student, one SP and two examiners. The mock session was video recorded as a demonstration of the system. Individual guides for students, examiners, SPs, proctors and administrators were also prepared and distributed in advance of the OSCE exams. An orientation session was also given to all participants. The system was used for end-of-year OSCE exams for medical students in years 3–6 of the MD program. Mock OSCEs were conducted before each of these examinations.

The OSCE time management website was developed using HTML5 for web page display, ASP.NET as the programming language, and MS SQL Server as the relational database for storing data. The system was developed by one programmer and one designer and took approximately 30 h of programming time to initially complete, although tweaks and enhancements to the program are ongoing as needed.

We conducted three major remote OSCEs using the system:

- Year 6 students (73) in two rounds with four circuits each and ten students per circuit. They had five history taking and communication stations with SPs and examiners, two viva stations with examiners only, and three rest stations.
- Year 3 students (80) in two rounds with four circuits each and ten students per circuit. They had six history taking and communication stations with SPs and examiners and four rest stations.
- Year 4 students (69) in two rounds with four circuits each and nine students per circuit. They had six history taking and communication stations with SPs and examiners and four rest stations.

A short survey was developed and distributed to participants seeking their opinions on training and use of the system. Institutional Review Board approval was obtained to carry out this survey using anonymous participant data (approval number: ERS_2020_6135).

The survey questions are shown below:

1. What was your role in the online OSCE? Student, Examiner, Simulated Patient, Proctor, Admin Staff.
2. Which MD year were you involved the online OSCE system? Year 3, Year 4, Year 5, Year 6.
3. The guide provided was helpful for me to understand how the system will work. (1) Strongly Disagree, (2) Disagree, (3) Neutral, (4) Agree, (5) Strongly agree.
4. The demonstration videos provided were helpful for me to understand how the system will work. (1) Strongly Disagree, (2) Disagree, (3) Neutral, (4) Agree, (5) Strongly agree.
5. The orientation session was helpful for me to understand how the system will work. (1) Strongly Disagree, (2) Disagree, (3) Neutral, (4) Agree, (5) Strongly agree.
6. During the OSCE exam, the Time Management System worked well to help with synchronized movement from station to station. (1) Strongly Disagree, (2) Disagree, (3) Neutral, (4) Agree, (5) Strongly agree.
7. Overall, I am satisfied with the OSCE Time Management System as a good way of synchronizing movement from station to station. (1) Strongly Disagree, (2) Disagree, (3) Neutral, (4) Agree, (5) Strongly agree.
8. How can the system be improved?
9. Please add any other comments you wish about OSCE online exam system:

We used a modified shorter version of the System Usability Scale (SUS) questionnaire [18] to evaluate these components. To obtain a good response, the questionnaire was reduced to five questions excluding two demographic questions at the beginning and two open-ended questions at the end. We did not pilot the questionnaire, but it was reviewed by several internal stakeholders of the OSCE examination.

An aggregate, anonymous comparison of student assessment marks was conducted between students examined previously in a physical setting (2018/2019) and these students examined online (2019/2020).

## Results

Based on the requirements analysis, the OSCE time management system was developed with the following user requirements in mind:

- An online system with secure login which allows users to view current station remaining time and upcoming station times.
- User access to the correct web page (view of the station list) based on the user type (student, examiner, simulated patient, proctor, and administrator).
- User access to that user’s appropriate rotation schedule with time for entry and link to virtual room.
- Display of station scenario at the start time and removal at the end time.
- Hands-free viewing of the website for students once logged in, with automatic refreshing of the webpage to indicate progress through the stations and time remaining in current station.
- Administrator view of OSCE exam progress with ability to delay start of next station for all users for a specified number of minutes.

The system was developed and tested based on these user requirements. A mock test was set up with one student, one SP and two examiners. The session was recorded as a demonstration for all users to view and understand how the system would work in real-time. Also, a written guide for each type of user was developed with images of the system to help them understand how the system works.

**Fig. 1:**
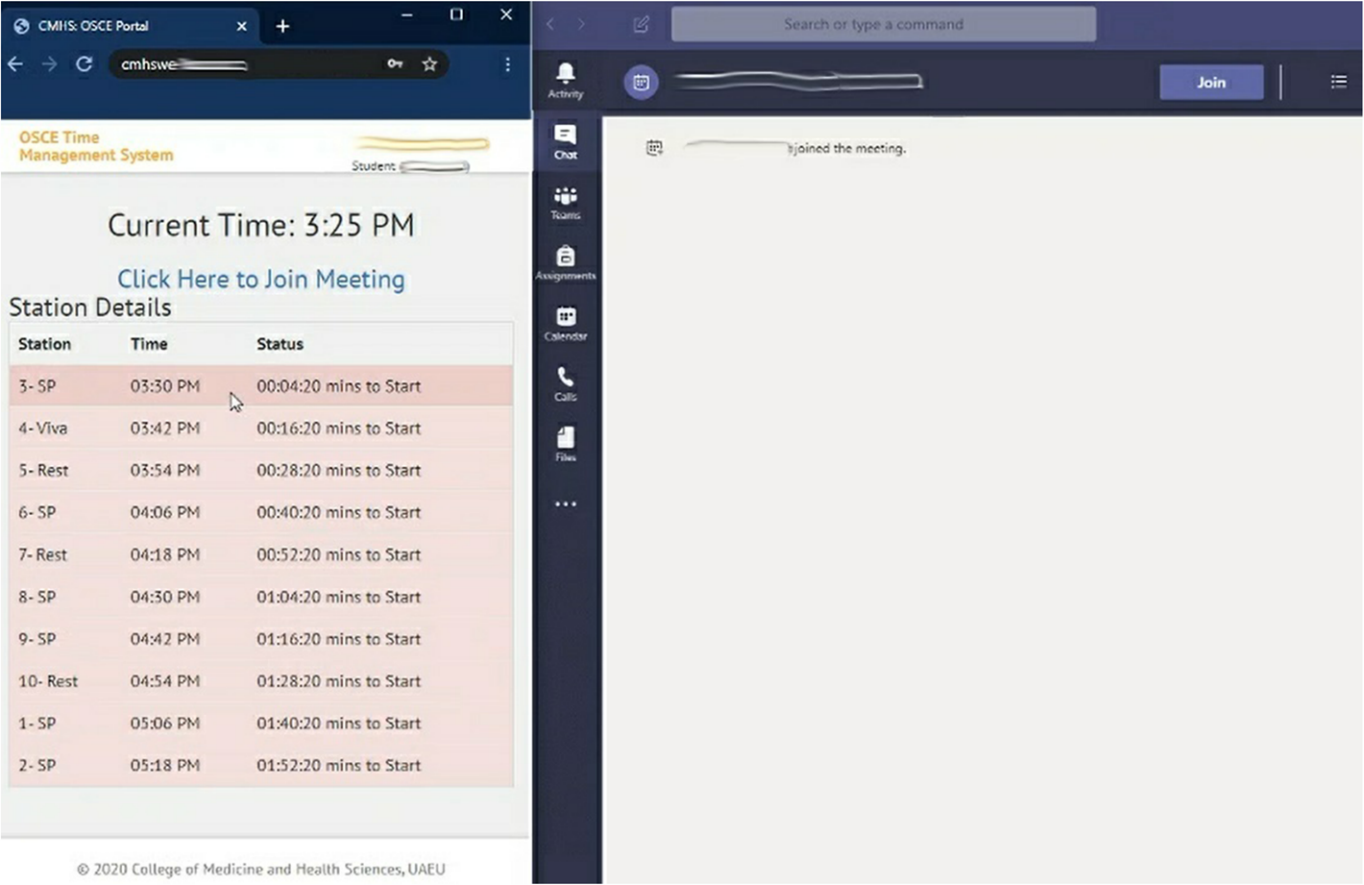
Student screen with the OSCE website on the left and Teams on the right (screenshot from the system)

**Fig. 2:**
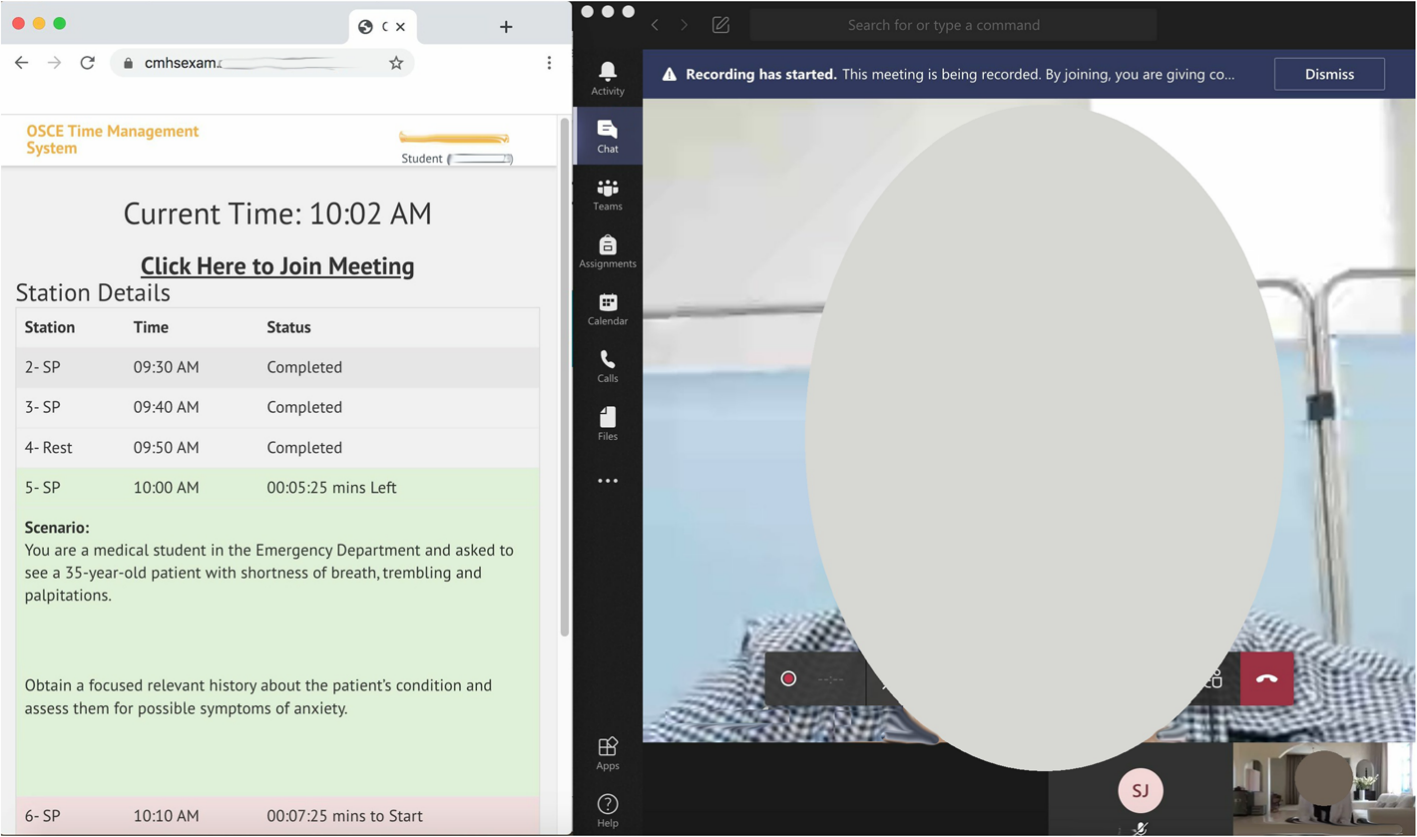
Student screen during one of the stations with examiner and SP also in the virtual room showing the OSCE time management system with the scenario and station time left (screenshot from the system and photo of the participates taken by authors)

We developed the following instructions for students:

1. Be ready in your remote, quiet and private location with your laptop connected to the Internet half an hour before the exam start time.
2. Log into the OSCE Time Management website: (link provided) using your University username and a password we will send you. Place the browser window on the left part of the screen. You may want to increase the font size on this website so that you can read the scenario from a meter away.
3. Log into Teams App using your University username and password, open it and place it on the right side of the screen taking up two thirds of the screen (Fig. 1).

4. Enter the Teams room designated for you on the link provided at the top of the OSCE Time Management website.
5. Connect the headset and sit a meter away from the computer.
6. Be ready half an hour before the exam start time for a proctor to enter your virtual room and test the light, audio, video and student’s seating far enough from the computer.
7. The Examiner and SP will enter the room at the start time, and you will see the stations scenario visible on the OSCE time management website at the start time as well (Fig. 2).

8. The OSCE Time Management website is your guide for when the station will start and end. It will refresh automatically every 10 s.
9. You will not need to approach the computer during the entire exam time as you will stay in this virtual room (with examiners and SPs entering and leaving for each station) and the OSCE website will refresh automatically letting you know when they will enter and when the station time will end.

**Fig. 3:**
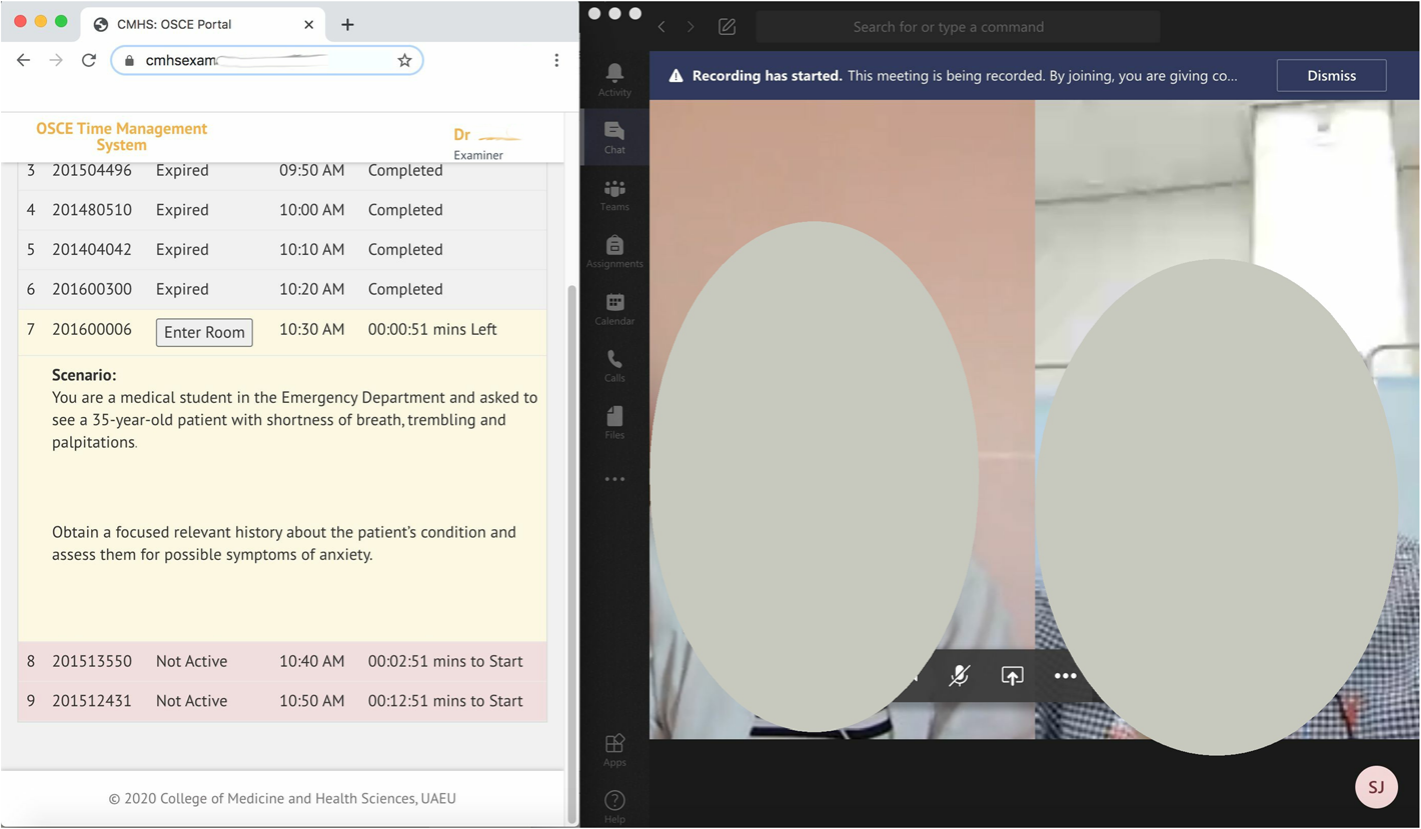
Examiner’s screen during the station with SP and student visible as well as OSCE time management system showing student name, station scenario and time remaining (screenshot from the system and photo of the participates taken by authors)

Similar instructions were developed for examiners, SPs and proctors. They also log into the OSCE time management system and use it as a guide for when to enter the virtual rooms (Fig. 3).

**Fig. 4:**
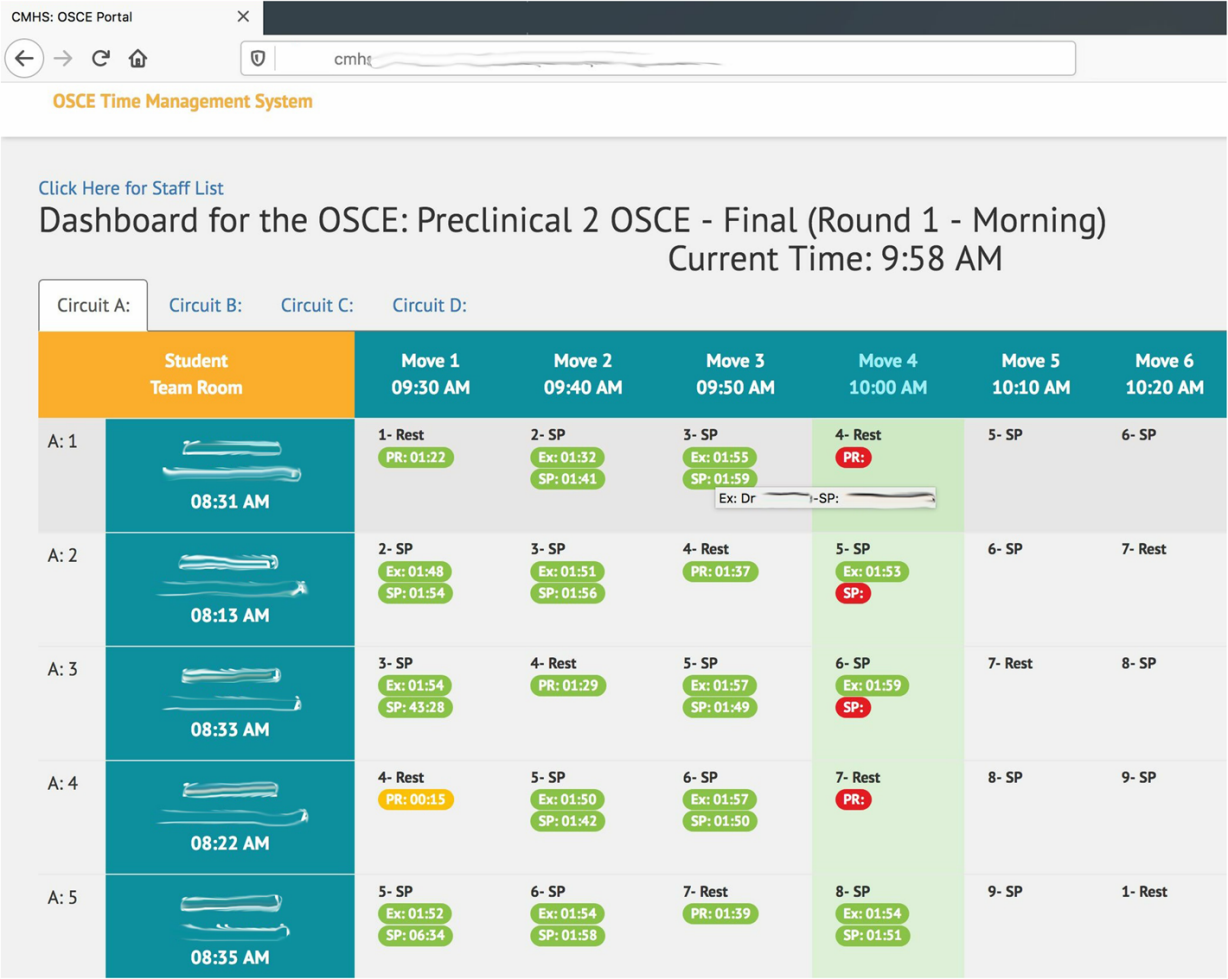
Administrator panel for the OSCE Time Management System showing exam progress in real-time (screenshot from the system)
- Have ability to delay start of next station for all users for a specified number of minutes. The delayed start time will appear synchronously for all participants.

For administration and IT overseeing exam progress, we also developed an admin panel on the website accessible to OSCE administrators and the IT team. They can:

- View time students, examiners, SPs and proctors have logged on and entered the virtual room.
- Enter the virtual room to check on the student and start recording.
- Have an overview of the progress as shown in Fig. 4. Color coding and mouse over functionality provide insightful information.

We conducted three OSCEs using this system as well as three short mock OSCEs for training. For the three OSCEs conducted there was a total of 6 rounds, 24 circuits, 236 students, 52 examiners, 50 SPs, 22 Proctors, and 2,332 movements between stations. For previous face-to-face Preclinical OSCEs there were eight stations and for the current remote OSCEs there were six stations. In the Clinical OSCE, there were twelve stations in prior years in contrast with seven stations for the remote OSCE in 2020.

### Satisfaction survey

The satisfaction survey was conducted after all three OSCE exams where complete and included 236 students, 52 examiners, 50 SPs and 22 Proctors for a total of 360 participant who were invited to take the survey. There were 187 respondents to the survey giving a response rate of 52 %. They were well distributed by type of user and student year in the program. Table 1 shows the high percentage of participants who chose ‘Agree’ or ‘Strongly Agree’ in response to selected survey questions.

**Table 1:** Percentage of participants who chose ‘Agree’ or ‘Strongly Agree’ in response to survey questions (n=187)

Survey Question	Percent choosing Agree or Strongly Agree
1. The written guide provided was helpful for me to understand how the system will work.	93.0 %
2. The demo videos provided were helpful for me to understand how the system will work.	85.0 %
3. The orientation session was helpful for me to understand how the system will work.	82.9 %
4. During the OSCE exam, the Time Management System worked well to help with synchronized movement from station to station.	88.2 %
5. Overall, I am satisfied with the OSCE Time Management System as a good way of synchronizing movement from station to station.	91.4 %

There was no difference in overall satisfaction with the system between the groups of participants: examiners, students and simulated patients (Kruskal-Wallis Test, *p* = 0.283).

Most open-ended comments were positive as illustrated by the following examples:

Student: *It was good and went smoothly, but it would be better if mock exams were always conducted before the actual test. Thank you for your great work and effort.*

Student: *It was surprisingly one of the best experiences.*

Student: *Thank you so much for your hard work. The system worked perfectly during the test. The system itself is really ingenious and well organized.*

Student: *I believe the MOCK OSCE was crucial and the single most important factor contributing to the success of the session.*

Examiner: *The system worked very well without much hitch.*

SP: *I suggest providing a laptop for SPs which will be helpful in synchronization from system to system.*

Proctor: *Force examiners and SPs to adhere to station time in order to avoid overlapping and confusion.*

### Examinee outcomes analysis

There was no significant difference in student marks in one of the OSCEs compared to the previous academic year (2018–2019) (independent t-test, *p =* 0.612), and in the other two OSCEs, marks this year (2020) were slightly higher than the previous academic year perhaps due to lack of physical exam stations.

## Discussion

In this study we developed and evaluated a high-stakes online OSCE incorporating a time-management web-based system to facilitate and synchronize examinee, examiner, SP and proctor movement from station to station. Given the differing techniques and resources necessitated for conducting high-stakes online OSCEs in challenging COVID-19 pandemic circumstances, we had a strong sense that purpose and context demanded focus on specific utility aspects of the assessment [17]. The discussion is therefore based on the objectives of this study and on assessment instrument utility indicators of feasibility, cost effectiveness, acceptability, and validity which are outlined below in comparison to traditional OSCEs and other online examples reported in the published literature.

### Implementation planning

Although the time management component of online OSCEs is new, the idea of online OSCEs that enable learners to demonstrate competencies and skills remotely has been performed previously [10, 12, 13]. Assessment of any kind requires careful planning and implementation of all components [17] and in the case of online OSCEs this was crucial to achieve quick and efficient restructuring of available resources. Factors contributing to successful implementation include early planning and active involvement of all stakeholders including students, faculty, simulated patients and support staff.

Planning is largely driven by ongoing review and quality assurance procedures such as blueprinting and mapping but there is obviously reduction in these processes when planning a seven station OSCE vs. a twelve station one. Detailed discussions regarding appropriate samples of skills-based outcomes amenable to remote assessment formed a substantial part of the planning phase. It was however, deemed impossible to include competency-based assessment of physical examination in our remote OSCE, rendering it a modified remote OSCE. To overcome potentially reduced content validity other health professions educators have suggested having students submit video-recorded physical examinations on high fidelity models [19].

Also, of importance is training and awareness of stakeholders through provision of clear guidelines and user manuals, conducting an extended pilot OSCE to test the developed software and to ensure user familiarity. Others observe the importance of good piloting of the system as the virtual environment itself may introduce barriers to assessment of appropriate patient care [20]. Three recent studies report steps and tips for running and managing remote OSCEs [15, 16, 20]. Good planning and implementation may help in identifying these barriers and the corrective measures [6, 21]. In summary, similar to most innovations, successful implementation relies on coordinating multiple activities, and doing so requires teamwork to assess available resources, creatively address additional resource needs, and evaluate the implementation for quality improvement purposes.

### Feasibility

Here, we consider the extent to which the online OSCE is practical, realistic, and sensible given the context and circumstances. Several authors describe practical steps required to design and run a successful traditional OSCE [1, 7, 20, 22] some indicating a need for large numbers of personnel under leadership of an assessment team. The online OSCE is no different and in fact requires additional IT personnel. The online OSCE format allows for the inclusion of most clinical skills stations, exceptions are the demonstration of practical skills which are not feasible due to the student and SP being in remote locations.

This OSCE implementation was quite feasible and practical in the sense that no extra efforts or logistics beyond the College facilities were needed. There were no problems with connectivity and all students had good access to the exam using normal WIFI connection within their homes. Examiners also experienced smooth flow of stations. This was enhanced with the time management system which gives an audio message 2 min before the station start, a signal 2 min before the allotted time for the station, and a final buzzer at the end of the station. This novel addition was not observed in other reported remote OSCE implementations which in contrast, utilized the usual manual bell [15, 16, 20, 23]. Also, a synchronized countdown timer is visible on the website for all participants in each station throughout the examination. In addition, the display of clear color coding of active and completed stations allows for a quick and user-friendly indication of where the participant is in the examination.

Evaluations from perspectives of examiners and students reveal the innovation provided an appropriate time-signaled environment for performance of skills which was practical and mimicked the traditional physical OSCE. This method is preferable to leaving responsibility for observing station length to students and examiners which is potentially anxiety provoking. However, in future to reduce confusion regarding what each buzzer signifies it might be useful to incorporate automated verbal commands such as “start station” “start feedback” and “end station”, etc.

### Cost effectiveness

Traditional OSCEs can be quite costly to set up in terms of human and physical resources [24]. The physical resources and equipment required for implementation of online OSCEs in terms of technology (computers with camera and microphone, and internet connection) were already provided by the institution. We used the same number of examiners and simulated patients and factored in similar logistic requirements as with traditional OSCEs. The addition of the time management system however took approximately 30 h from one full time programmer to develop and test. Students used their own devices and regular home connections. One study reported minimal costs of developing a virtual OSCE program using videoconferencing technology utilized in their remote OSCE in 2015 [13]. No cost figures were stated but as a collaboration between the colleges of Nursing and Medicine, the operation of this virtual OSCE program was achieved with minimal financial impact.

### Usability, acceptance and satisfaction

Usability refers to the software’s ability to be understood, learned, operated and attractive to the user, when used under specified conditions [21], while acceptability and satisfaction refers to the extent to which process and outcomes of system was acceptable to users and whether they found results of the process and outcomes credible.

We used a modified shorter version of System Usability Scale (SUS) questionnaire to evaluate these components. To obtain a good response, the questionnaire was reduced to five questions not including two demographic questions in the beginning and two open-ended questions at the end. Results of the survey for both students and examiners showed high levels of usability of 84 % which is comparatively higher than the acceptable level of 68.5 % [18] and good level of usability of 73 % [25, 26]. Acceptability is also high as evident from the positive qualitative responses such as, “*It was surprisingly one of the best experiences*”. The few negative comments observed were regarding adhering to time by examiners and providing laptops to simulated patients. The high levels of usability and acceptability are comparable to a number of similar recent studies [10, 11].

### Validity and reproducibility

Validity is a property of the application of the assessment and in concurrence with Hodges [3], we believe a relevant applicable question here is “For whom and in what circumstances might a particular OSCE produce results that reflect a valid assessment of competence?” [22]. We therefore, consider whether the content and results of the online OSCE assessment were appropriate for the particular purpose and context in which it was used. The purpose of the online OSCE was to specifically assess history taking and communication and professionalism skills. In contrast, traditional OSCEs assess a wider range of skills including physical examination. There are obvious challenges to authentically assessing physical examination skills in an online OSCE which could impact how well the assessment extrapolates to real world clinical performance. Other authors suggest however, that the more focused the OSCE blueprint the better it will provide validity evidence for generalization to other similar assessment settings; though at the expense of extrapolation to other skills [27, 28]. This presents another unanswered question as to where else could a wider range of performance and clinical reasoning skills be assessed under the current COVID-19 circumstances in which undergraduate medical students continue to have little or no access to traditional OSCE test settings and clinical environments.

We estimated the content or face validity of our OSCE through a committee of experts representing all clinical departments and medical education. All agreed that with the exception of physical examination skills, most of the competencies tested in our traditional OSCEs were represented in this remote one. This limitation of online OSCEs cannot however be overlooked, as physical examination cannot be measured remotely without physical access to simulated or real patients. To overcome the limitation, other authors suggest integrating stations where students have opportunity to verbalize physical examination steps following demonstration by the examiner. Even this alternative does not give students opportunity for actual performance of the task although other authors found that it was possible to develop a prototype for use in the assessment of clinical skills including physical examination using computer graphics technology combining virtual simulators [26]. They assert that this prototype maintains characteristics typical of the traditional OSCE. However, in a competency based curriculum, this method may not include a good sampling of physical exam encounters. Further research is recommended in this area to suggest approaches that are more authentic and valid.

The reproducibility or reliability of scores is an important component of validity evidence. Reliability and validity of OSCEs are also influenced by number of stations and length of the examination [28, 29]. Cronbach’s alpha or Generalizability G value are often used to measure overall reliability of traditional OSCEs [28]. The Cronbach’s alpha for these online exams were comparable with similar components of traditional OSCEs conducted previously at our institution.

This innovation is relevant to all levels of health professions education that may have a need to use online OSCEs. We believe that the technology involved in the system is very basic (simply a self-refreshing webpage) and would not have impacted student’s ability to use the system. Additionally, faculty, SPs and students received demonstration-type training beforehand.

## Conclusions

It is possible to develop and use a virtual OSCE supplemented with a time management system with specifications that demonstrate utility for conducting online OSCEs from the perspectives of relevant groups of stakeholders. The system is cost effective as it is developed in-house in a short amount of time utilizing available human and physical resources. The system is earnestly accepted by all participants, and student results are not dissimilar to traditional OSCEs. In the mid 1970 s Harden et al. [1] introduced the traditional OSCE to overcome perceived deficiencies of the traditional oral clinical examination; in Spring 2020 in the context of challenges dictated by the COVID-19 pandemic, we developed a web-based time-management system for high-stakes online OSCEs to overcome logistical difficulties including the need for examiners, SPs and proctors to move through stations in a coordinated fashion, that is feasible, cost-effective, acceptable and valid. A future challenge is to develop online OSCEs that authentically test physical examination skills.

### Recommendations

- Develop clear audio-visual and written guides which include illustrative images for each of the participating groups explaining how the system will work.
- Conduct a mock OSCE for each group of participants as it is necessary to ensure good understanding of how the system will work.
- Those who have online OSCE needs are recommended to develop a similar system, search for one that meets the requirements, or contact the authors as this web system may become available for public use. The usage guide and demonstration video are also available upon request.
- Where possible, collaboration with other health professions education institutions or those using telehealth systems may achieve cost reductions in sourcing appropriate systems.

### Limitations

Physical exam stations are not possible in an online setting as the student and SP must be in the same location for a physical exam. However, it is possible for the examiner to ask questions about how a student may conduct portions of the physical exam which may satisfy this assessment component. This study was conducted in a single medical school using three OSCE exams of three groups of students in the same academic year, and a single time point survey regarding user satisfaction. The generalizability to other settings may be limited. The need for further larger studies are indicated.

## Data Availability

The datasets used and/or analyzed during the current study are available from the corresponding author on reasonable request.

## Abbreviations

OSCEs: Objective Structured Clinical Examinations.
SPs: Simulated Patients
SUS: System Usability Scale
